# Follow-up of a 10-year period (2010-2020) of Down syndrome in Brazil

**DOI:** 10.1590/1807-3107bor-2025.vol39.090

**Published:** 2025-09-08

**Authors:** Roberta Magalhães MIRANDA, Joanna Lara Saraiva de PAULA, Thiago Rezende dos SANTOS, Rafaela da Silveira PINTO, Soraia MACARI

**Affiliations:** (a) Universidade Federal de Minas Gerais – UFMG, School of Dentistry, Department of Restorative Dentistry, Belo Horizonte, MG, Brazil.; (b) Universidade Federal de Minas Gerais – UFMG, Institute of Exact Sciences Department of Statistics, Belo Horizonte, MG, Brazil.; (c) Universidade Federal de Minas Gerais – UFMG, School of Dentistry, Department of Social and Preventive Dentistry, Belo Horizonte, MG, Brazil.

**Keywords:** Down Syndrome, Epidemiology, Follow up Studies, Epidemiologic Surveillance Services, Health Information Systems

## Abstract

This study aimed to determine the prevalence and provide an overview of Down syndrome and child- and mother-associated factors in Brazil from 2010 to 2020. This was a cross-sectional study including epidemiological characteristics related to live births of individuals with and without Down syndrome using the Brazilian government website. The average prevalence of Down syndrome in Brazil was approximately 30.4 children per 100,000 live births during the 10-year period, corresponding to 1 case in every 3,289.47 newborns. The occurrence of Down syndrome was increased in White children, preterm births, and low birth weight infants compared to the no-Down syndrome group; however, it was decreased in males. The prevalence of Down syndrome was low among mothers without a spouse, insufficient prenatal care, and vaginal delivery. Conversely, the prevalence was high among mothers aged 35 years and older and mothers considered White. There was no evidence of a time correlation in the prevalence among the regions of Brazil during this 10-year period. It is imperative to strengthen national monitoring of the prevalence of children with Down syndrome and provide better support to mothers and public services for this group.

## Introduction

Trisomy 21, also known as Down syndrome, is a chromosomal disorder caused by the presence of a supernumerary chromosome 21. The pathophysiology of Down syndrome is related to an extra copy of chromosome 21, which occurs because chromosome 21 does not segregate during gametogenesis; therefore, all somatic cells are affected.^1^ However, depending on the patient’s karyotype (e.g., mosaicism), some cells may have an extra chromosome 21, whereas others may not.^2^ It is the most common congenital cause of intellectual disability and presents with signs of chronic immune system dysregulation, including a high prevalence of autoimmune disorders, leading to numerous metabolic and structural problems.^3,4^ Additionally, they present with growth retardation, neurological features, congenital heart defects, gastrointestinal abnormalities, and characteristic facial features.^5,6^

The frequency of Down syndrome is not known; however, the incidence of the disorder increases markedly with increasing maternal age, and there is some evidence that paternal age may also play a role.^7,8^ Most men with Down syndrome are infertile. There are only two confirmed reports of paternity in individuals without mosaicism, but there are numerous reports of successful pregnancies in women.^9^

The incidence of Down syndrome differs by population worldwide.^3,10-12^ In Brazil, data on the incidence of babies with Down syndrome remain unclear. Brazilian studies often use small samples, based mostly on hospital statistics or a few locations in the country.^11^ In the early 1990s, the Brazilian Information System for Live Births (Sinasc in Portuguese) was created by the Unified Health System (SUS in Portuguese) and implemented nationwide. These database systems have improved the control of the national analysis of life events by developing reliable demographic indicators of health data.^13^

The aim of this study was to describe the history of a 10-year period (2010–2020), current prevalence, and relationship between the presence of Down syndrome and factors related to the mother (e.g., age, ethnicity, marital status, schooling, and prenatal care) and child (e.g., birth weight, ethnicity, sex, and preterm birth) in Brazil and by state.

## Methods

### Data Source

This epidemiological study used records from the Brazilian government website with the help of the Brazilian Public Health System Computer Department (Datasus) of the Brazilian Ministry of Health. Ethical evaluation was not required because data were obtained from secondary databases.

### Prevalence Rate

Sinasc was used to determine the prevalence of newborns with Down syndrome during the 10-year period from 2010 to 2020 involving the 27 states of Brazil. The prevalence rate of Down syndrome was calculated by dividing the number of live births with Down syndrome by the mother’s place of residence by the total number of live births in the same place and during the same period, multiplied by 100,000 inhabitants, and measuring the average change (95% confidence interval [CI]).^14^

### Prevalence Rate with and without Down syndrome in the period 2010–2020

The Sinasc was also used to extract information regarding the associated factors of newborns and maternal socioeconomic conditions and demographic characteristics for the period 2010-2020. These variables were ranked by the proportion of live births with and without Down syndrome (no Down syndrome) under the following conditions: a) infants with low birth weight (up to 2500 g), b) sex (male), c) skin color/race according to the genetic background of the Brazilian population (individuals classified as White) ^15^ of the newborn, and d) preterm birth (birth at gestational age less than 37 weeks). The variables analyzed regarding the mothers included schooling up to the 8th grade, maternal marital status (single, divorced, or widowed), aged 35 years or older, mothers classified as white race, inadequate prenatal care (less than seven consultations), and vaginal delivery.

### Proportion of municipalities with cases of Down syndrome in the period 2010-2020

The proportion of municipalities with cases of Down syndrome was calculated by dividing the number of municipalities in each state that had at least one case of Down syndrome from 2010 to 2020 by the total number of municipalities in that state.

### Statistical Analysis

The databases, including estimated point of prevalence rate and confidence interval (CI), were organized using Excel 365® software (Microsoft Office 365, Redmond, USA). Statistical analysis was performed using the Statistical Package for the Social Sciences (SPSS for Windows, version 23.0; SPSS Inc., Chicago, USA). Logistic regression and normal models were used to analyze the data, and the explanatory variables of the region were included in the models. The dependent variable in the logistic regression was the presence of Down syndrome, which is a binary variable. The Midwest region was selected as the reference category in the logistic regression analysis. However, any of the five regions could have been chosen as the base category if the comparison between the categories was of great importance. Moran’s test was performed to evaluate the presence of spatial correlations. p < 0.05 was considered statistically significant.

## Results

According to the data obtained from SINASC, 9,692 births of children with Down syndrome were recorded in Brazil between 2010 and 2020. The average prevalence of Down syndrome in Brazil was approximately 30 per 100,000 live births, which corresponds to 1 child for approximately every 3,289.47 live births in this period (Table 1). Regarding the latest update in January 2022, Brazil had 5,575 municipalities. The number and percentage of municipalities in Brazil with at least one case of Down syndrome in Brazil in the period from 2010 to 2020 was 2,123 and 38.1%, respectively. This implies that almost two-fifths of the Brazilian municipalities had at least one case of Down syndrome, independent of the ethnic groups.

**Table 1:** Number and percentage of municipalities with at least one case of Down syndrome and the prevalence of live births with Down syndrome per 100,000 live births from 2010 to 2020 by states.

States (abbreviation)/region	Number and percentage of municipalities with at least one case of Down Syndrome from 2010 to 2020	Prevalence of live births with Down Syndrome per 100,000 live births from 2010 to 2020
North region		
Rondônia (RO)	15 (28.8)	19.5
Acre (AC)	15 (68.2)	32.9
Amazonas (AM)	36 (58.1)	20.8
Roraima (RR)	10 (66.7)	26.5
Pará (PA)	80 (55.6)	17.8
Amapá (AP)	9 (56.3)	36.6
Tocantins (TO)	24 (17.3)	16.6
Northeast region		
Maranhão (MA)	63 (29.0)	11.6
Piauí (PI)	30 (13.4)	12.1
Ceará (CE)	119 (64.7)	38.2
Rio Grande do Norte (RN)	55 (32.9)	31.5
Paraíba (PB)	76 (34.1)	29.9
Pernambuco (PE)	106 (57.3)	27
Alagoas (AL)	52 (51.0)	24.9
Sergipe (SE)	41 (54.7)	41.5
Bahia (BA)	130 (31.2)	17.7
Southeast region		
Minas Gerais (MG)	232 (27.2)	26.9
Espírito Santo (ES)	41 (52.6)	36.7
Rio de Janeiro (RJ)	61 (66.3)	26.2
São Paulo (SP)	317 (49.1)	42
South region		
Paraná (PR)	166 (41.6)	36.6
Santa Catarina	136 (46.1)	49.8
Rio Grande do Sul (RS)	175 (35.1)	47.8
Midwest region		
Mato Grosso do Sul (MS)	24 (30.4)	17.8
Mato Grosso (MT)	47 (33.3)	22.2
Goiás (GO)	63 (25.6)	16.5
Federal District (DF)	1 (100 .0)	21.9
Brazil	2,123 (38.1)	30.4

Source: Sinasc.

The 10-year study period revealed the prevalence of Down syndrome in various regions of Brazil. The lowest and highest prevalence between 2010 and 2020 in the northern region were 13.98 (year 2016) and 24.75 (year 2018) per 100,000 live births, respectively; Northeast region were 22.34 (year 2012) and 27.22 (year 2010) per 100,000 live births, respectively; Southeast region were 29.08 (year 2014) and 41.33 (year 2020) per 100,000 live births, respectively; South region were 36.24 (year 2016) and 54.75 (year 2011) per 100,000 live births, respectively; and Midwest region were 11.08 (year 2013) and 27.77 (year 2020) per 100,000 live births, respectively (Figure 1). Furthermore, there was no evidence of a time correlation among the various regions of Brazil (Figure). The analysis of the results of the logistic regression binomial model and considering the Midwest region as a reference category/class, the North, Northeast, South, and Southeast regions were not significantly associated with the prevalence of Down syndrome (Figure). The only significant explanatory variables were male children and the proportion of preterm births with odds ratio of 0.73 (1.38) and 1.82 (0.55) of having Down syndrome, respectively.

**Figure:**
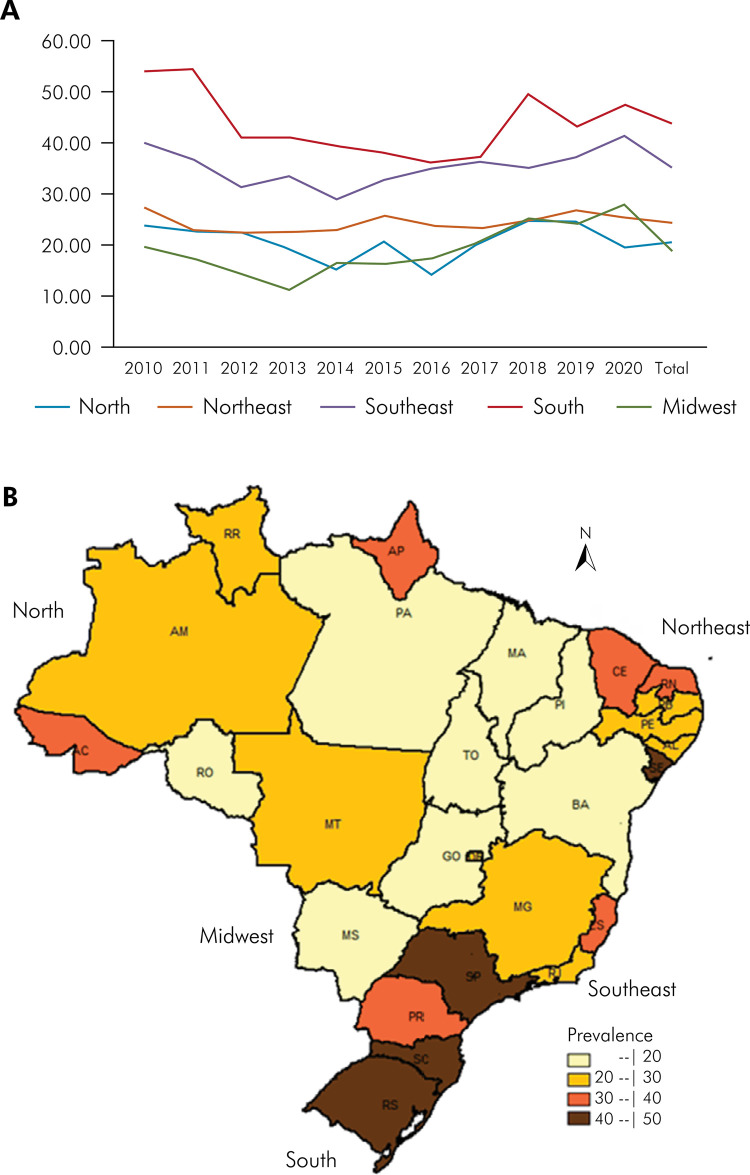
A. Prevalence of babies with Down syndrome per 100,000 live births by regions in Brazil between 2010 and 2020. B. Distribution of the average prevalence of babies with Down syndrome per 100,000 live births from 2010 to 2020.

Regarding the prevalence rates during the 10-year period in the states of Brazil, Santa Catarina had the highest prevalence, followed by Rio Grande do Sul, both located in the southern region. São Paulo in the Southeast region and Sergipe in the Northeast region had the third and fourth highest prevalence, respectively. The prevalence in the northern states in ascending order were Tocantins, Para, Rondônia, Amazonas, Roraima, Acre, and Amapá. Concerning the prevalence of estimated cases in various municipalities in 2020, the state with the highest number of cases was Acre, followed by Roraima and Rio de Janeiro (Table 1 and Figure).

At the national level in Brazil, children with Down syndrome were more likely to have low birth weights, white children, and preterm births than newborns without Down syndrome. However, the proportion of male children with Down syndrome was lower than those without Down syndrome (Table 2). Although the incidence of Down syndrome among children of African descent was slightly higher (51.21%) than that among white children with Down syndrome (48.79%), the proportion of children with Down syndrome to children without Down syndrome was lower in children of African descent than those of white descent (Table 2).

**Table 2:** Prevalence of children with Down syndrome according to birth weight, ethnicity, gender, and preterm birth from 2010 to 2020 by states in Brazil.

Variables	Children with low-birth weight (up to 2.500 grams)		Children classified as White		Male children		Proportion of preterm births (up to 37 weeks)	
Federative unit (Abbreviation)	Down %	No Down %	Down %	No Down %	Down %	No Down %	Down %	No Down %
	(CI95%)	(CI95%)	(CI95%)	(CI95%)	(CI95%)	(CI95%)	(CI95%)	(CI95%)
	p-value		p-value		p-value		p-value	
North Region								
Rondônia (RO)	20.68	6.82	15.51	24.30	39.65	51.33	8.62	8.95
	(9.68–31.7)	(0.00–13.67)	(5.68–25.36)	(12.64–35.96)	( 26.36–52.96 )	( 37.75–64.93 )	( 0.99–16.25 )	( 1.19–16.71 )
	p <0.05		p > 0.05		p > 0.05		p > 0.05	
Acre (AC)	23.33	7.51	6.67	7.37	45	51.45	33.33	12.12
	(0.0–52.3)	(1.9–10.8)	(73.9–100.0)	(78.9–97.9)	(0.0–100.0)	(49.1–56.4)	(0.0–100.0)	(7.2–17.2)
	p < 0.05		p > 0.05		p > 0.05		p < 0.05	
Amazonas (AM)	17.98	7.5	8.99	6.33	43.82	51.21	17.98	11.05
	(5.66–41)	(0.00–18.54)	(0.00–17.1)	(0.00–18.29)	( 24.21–65.79 )	( 30.57–72.33 )	( 13.63–53.03 )	( 0.00–25.76 )
	p >0.05		p > 0.05		p > 0.05		p > 0.05	
Roraima (RR)	20.59	8.18	5.88	7.98	41.18	51.4	8.82	13.65
	(0.13–41.05)	(0.00–22.05)	(0.00–17.79)	(0.00–21.69)	( 16.27–66.09 )	( 26.11–76.69 )	( 0.00–23.17 )	( 0.00–31.02 )
	p > 0.05		p > 0.05		p > 0.05		p > 0.05	
Pará (PA)	18.68	7.54	8.06	7.46	46.52	51.29	17.22	11.11
	(12.31–25.05)	(3.23–11.85)	(3.61–12.51)	(3.17–11.75)	( 38.37–54.67 )	( 43.13–59.45 )	( 11.05–23.39 )	( 5.98–16.24 )
	p <0.05		p > 0.05		p > 0.05		p > 0.05	
Amapá (AP)	12.9	8.2	11.29	10.18	58.06	51.27	6.45	12.22
	(0.00–29.32)	(0.00–21.64)	(0.00–26.8)	(0.00–25)	( 33.88–82.24 )	( 26.78–75.76 )	( 0.00–18.49 )	( 0.00–28.27 )
	p < 0.05		p > 0.05		p > 0.05		p > 0.05	
Tocantins (TO)	20	7.42	20	13.4	66.67	51.2	24.44	10.65
	(13.35–26.65)	(3.06–11.78)	(13.35–26.65)	(7.74–19.06)	( 58.83–74.51 )	( 42.89–59.51 )	( 17.3–31.58 )	( 5.52–15.78 )
	p <0.05		p > 0.05		p < 0.05		p < 0.05	
Northeast Region								
Maranhão (MA)	18.37	7.32	13.61	8.57	36.05	51.33	21.09	10.21
	(13.22–23.52)	(3.85–10.79)	(9.05–18.17)	(4.85–12.29)	( 29.66–42.44 )	( 44.68–57.98 )	( 15.66–26.52 )	( 6.18–14.24 )
	p < 0.05		p > 0.05		p < 0.05		p < 0.05	
Piauí (PI)	34.38	7.72	3.13	11.33	40.63	51.19	26.56	10.19
	(28.16–40.6)	(4.22–11.22)	(0.85–5.41)	(7.18–15.48)	( 34.2–47.06 )	( 44.64–57.74 )	( 20.78–32.34 )	( 6.23–14.15 )
	p < 0.05		p < 0.05		p < 0.05		p < 0.05	
Ceará (CE)	19.33	8.03	8.36	9.08	48.51	51.26	21.93	11.08
	(13.62–25.04)	(4.1–11.96)	(4.36–12.36)	(4.93–13.23)	( 41.29–55.73 )	( 44.04–58.48 )	( 15.95–27.91 )	( 6.54–15.62 )
	p < 0.05		p > 0.05		p > 0.05		p < 0.05	
Rio Grande do Norte (RN)	15.43	8.25	40.74	29.99	48.15	51.19	18.52	12.27
	(9.95–20.91)	(4.08–12.42)	(33.29–48.19)	(23.04–36.94)	( 40.57–55.73 )	( 43.61–58.77 )	( 12.63–24.41 )	( 7.29–17.25 )
	p < 0.05		p < 0.05		p > 0.05		p > 0.05	
Paraíba (PB)	19.47	7.22	13.16	13.86	49.47	51.24	24.21	10.41
	(14.27–24.67)	(3.82–10.62)	(8.72–17.6)	(9.32–18.4)	( 42.91–56.03 )	( 44.68–57.8 )	( 18.59–29.83 )	( 6.4–14.42 )
	p < 0.05		p > 0.05		p > 0.05		p < 0.05	
Pernambuco (PE)	19.8	7.79	28.12	21.11	41.32	51.16	18.83	10.8
	(14.04–25.56)	(3.92–11.66)	(21.62–34.62)	(15.21–27.01)	( 34.21–48.43 )	( 43.94–58.38 )	( 13.18–24.48 )	( 6.32–15.28 )
	p <0.05		p > 0.05		p > 0.05		p < 0.05	
Alagoas (AL)	17.02	7.74	8.51	7.66	44.68	51.19	24.82	10.22
	(9.73–24.31)	(2.55–12.93)	(3.09–13.93)	(2.5–12.82)	( 35.03–54.33 )	( 41.49–60.89 )	( 16.44–33.2 )	( 4.34–16.1 )
	p < 0.05		p > 0.05		p > 0.05		p < 0.05	
Sergipe (SE)	16.23	8.06	15.58	10.49	43.51	51.3	14.94	9.39
	(7.89–24.57)	(1.9–14.22)	(7.37–23.79)	(3.56–17.42)	( 32.29–54.73 )	( 39.99–62.61 )	( 6.87–23.01 )	( 2.79–15.99 )
	p > 0.05		p > 0.05		p > 0.05		p > 0.05	
Bahia (BA)	21.16	8.44	9.57	8.16	46.1	51.15	20.4	10.22
	( 17.24–25.08)	( 5.77–11.11)	( 6.75–12.39)	( 5.53–10.79)	( 41.32–50.88 )	( 46.35–55.95 )	( 16.53–24.27 )	( 7.31–13.13 )
	p < 0.05		p > 0.05		p > 0.05		p < 0.05	
Southeast Region								
Minas Gerais (MG)	23.6	9.37	45.82	34.49	47.59	51.13	25.03	10.76
	( 20.75–26.45)	( 7.41–11.33)	( 42.48–49.16)	( 31.3–37.68)	( 44.24–50.94 )	( 47.78–54.48 )	( 22.12–27.94 )	( 8.68–12.84 )
	p < 0.05		p < 0.05		p > 0.05		p < 0.05	
Espírito Santo (ES)	21.82	7.98	39.55	27.09	49.09	51.33	25.91	9.33
	( 12.65–30.99)	( 1.97–13.99)	( 28.7–50.4)	( 17.23–36.95)	( 38–60.18 )	( 40.24–62.42 )	( 16.19–35.63 )	( 2.88–15.78 )
	p < 0.05		p > 0.05		p > 0.05		p < 0.05	
Rio de Janeiro (RJ)	20.79	9.13	46.45	37.01	47.56	51.1	20	10.78
	( 12.5–29.08)	( 3.24–15.02)	( 36.26–56.64)	( 27.14–46.88)	( 37.36–57.76 )	( 40.89–61.31 )	( 11.83–28.17 )	( 4.44–17.12 )
	p < 0.05		p > 0.05		p > 0.05		p > 0.05	
São Paulo (SP)	25.27	9.2	66.46	60.04	49.25	51.21	26.27	10.93
	( 21.92–28.62)	( 6.97–11.43)	( 62.82–70.1)	( 56.26–63.82)	( 45.39–53.11 )	( 47.35–55.07 )	( 22.87–29.67 )	( 8.52–13.34 )
	p < 0.05		p < 0.05		p > 0.05		p < 0.05	
South Region								
Paraná (PR)	23.43	8.51	81.38	78.41	48.15	51.24	20.87	9.93
	( 19.27–27.59)	( 5.77–11.25)	( 77.56–85.2)	( 74.37–82.45)	( 43.25–53.05 )	( 46.34–56.14 )	( 16.88–24.86 )	( 7–12.86 )
	p < 0.05		p > 0.05		p > 0.05		p < 0.05	
Santa Catarina (SC)	22.81	7.87	87.84	86.05	47.95	51.33	27.68	10.37
	( 18.02–27.6)	( 4.8–10.94)	( 84.11–91.57)	( 82.1–90)	( 42.25–53.65 )	( 45.63–57.03 )	( 22.57–32.79 )	( 6.89–13.85 )
	p < 0.05		p > 0.05		p > 0.05		p < 0.05	
Rio Grande do Sul (RS)	22.95	9.35	84.02	82.74	52.46	51.29	24.18	11.58
	( 19.25–26.65)	( 6.79–11.91)	( 80.8–87.24)	( 79.42–86.06)	( 48.07–56.85 )	( 46.9–55.68 )	( 20.42–27.94 )	( 8.77–14.39 )
	p < 0.05		p > 0.05		p > 0.05		p < 0.05	
Midwest Region								
Mato Grosso do Sul (MS)	23.81	7.91	36.9	37.97	38.1	51.19	22.62	11.13
	( 14.42–33.2)	( 1.96–13.86)	( 26.26–47.54)	( 27.27–48.67)	( 27.39–48.81 )	( 40.17–62.21 )	( 13.39–31.85 )	( 4.19–18.07 )
	p < 0.05		p > 0.05		p > 0.05		p = 0.05	
Mato Grosso (MT)	17.91	7.48	25.37	25.67	47.76	51.2	21.64	10.29
	( 11.58–24.24)	( 3.14–11.82)	( 18.19–32.55)	( 18.46–32.88)	( 39.52–56 )	( 42.95–59.45 )	( 14.84–28.44 )	( 5.28–15.3 )
	p < 0.05		p > 0.05		p > 0.05		p < 0.05	
Goiás (GO)	17.92	8.39	30.81	27.75	51.45	51.1	17.34	10.02
	( 13.13–22.71)	( 4.93–11.85)	( 25.04–36.58)	( 22.15–33.35)	( 45.2–57.7 )	( 44.85–57.35 )	( 12.61–22.07 )	( 6.27–13.77 )
	p < 0.05		p > 0.05		p > 0.05		p < 0.05	
Federal District (DF)	20	9.52	30.48	23.29	51.43	51.12	16.19	10.96
	*	*	*	*	*	*	*	*
	*	*	*	*	*	*	*	*
Brazil	22.25	8.49	48.79	36.71	47.97	51.21	23.05	10.7
	( 21.16–23.34)	( 7.76–9.22)	( 47.48–50.1)	( 35.44–37.98)	( 46.66–49.28 )	( 49.9–52.52 )	( 21.94–24.16 )	( 9.89–11.51 )
	p < 0.05		p < 0.05		p < 0.05		p < 0.05	

*Confidence interval 95% not calculated because the values are constant (a single municipality); Down: individuals with Down syndrome; No Down: individuals without Down syndrome. p < 0.05. Z-test was used for comparison between the Down and No Down groups.

Source: SINASC.

Significant differences were also observed between states in Brazil (Table 2). A high prevalence of newborns with Down syndrome and low birth weight was observed in all states, except for Amazonas and Roraima in the northern region, Sergipe in the Northeast region, and Mato Grosso do Sul in the Center-West region. Considering the ethnicity (children classified as White) of the newborns, a high prevalence of children with Down syndrome were observed in Piauí and Rio Grande do Norte in the Northeast Region and Minas Gerais and São Paulo in Southeast region (Table 2).

Regarding gender, the prevalence of Down syndrome in males was significantly high in the state of Tocantins in the North region and both Maranhão and Piauí in the Northeast region (Table 2). The states of Acre and Tocantins had an increased proportion of births of children with Down syndrome with a gestational age of less than 37 weeks. However, there was no significant difference in all other states of the northern region. The incidence of Down syndrome with preterm birth was significantly high in all other states of Brazil, except in Rio Grande do Norte and Sergipe in the Northeast region, Rio de Janeiro in the Southeast region, and Mato Grosso do Sul, in the Center-West region (Table 2).

The socioeconomic profile of mothers in Brazil showed significant differences at the national level. The prevalence of Down syndrome was lower compared to that of no Down syndrome in mothers without a spouse. The prevalence of Down syndrome was high among mothers aged 35 years and older and mothers considered as White (Table 3). At the state level, there was a significantly lower prevalence of Down syndrome compared to that with no Down syndrome among mothers with up to 8 years of schooling in Tocantins in the northern region (Table 3). Regarding mothers without a spouse, the prevalence of Down syndrome was low in the states of Maranhão and Bahia in the Northeast region, Minas Gerais and São Paulo in the Southeast region, and Rio Grande do Sul in the South region (Table 3). The prevalence of Down syndrome was significantly high in mothers aged 35 years and older in all states except in Mato Grosso in the Central-west region (Table 3). Concerning the mother’s ethnicity, the prevalence of Down syndrome was low in the state of Piauí in the Northeast region, whereas it was high in the state of Minas Gerais in Southeast region (Table 3). The prevalence of Down syndrome among mothers of Afro-descent was slightly higher compared to white children with Down syndrome; however, the proportion of live-born children with Down syndrome to no Down syndrome was lower in Afro-descent mothers than in those of white descent.

**Table 3:** Prevalence of sociodemographic profile of the Mother from 2010 to 2020 by states in Brazil.

Federative unit (Abbreviation)/Region	Mothers with up to 8 years of schooling		Mothers without a spouse present (single. divorced. or widowed)		Mothers aged 35 and older		Mothers classified as White	
	Down %	No Down %	Down %	No Down %	Down %	No Down %	Down %	No Down %
	(CI95%)	(CI95%)	(CI95%)	(CI95%)	(CI95%)	(CI95%)	(CI95%)	(CI95%)
	*p-value*		*p-value*		*p-value*		*p-value*	
North Region								
Rondônia (RO)	25.86	27.74	43.1	36.28	39.66	8.85	8.62	18.82
	(13.96–37.76)	(15.57–39.91)	(29.64–56.66)	(23.21–49.35)	(26.36–52.96)	(1.13–16.57)	(1.00–16.25)	(8.20–29.44)
	p > 0.05		p > 0.05		p < 0.05		p > 0.05	
Acre (AC)	41.67	41.07	30	25.01	55	10.4	6.67	6.38
	(21.07–62.27)	(20.51–61.63)	(10.85–49.15)	(6.91–43.10)	(34.21–75.78)	(0.00–23.16)	(0.00–17.10)	(0.00–16.59)
	p > 0.05		p > 0.05		p < 0.05		p > 0.05	
Amazonas (AM)	40.45	30.79	50.56	60.29	48.88	9.62	5.62	5
	(28.23–52.63)	(30.2–40.9)	(38.12–63.00)	(48.11–72.47)	(36.44–61.32)	(2.28–16.96)	(0.00–11.35)	(0.00–10.42)
	p > 0.05		p > 0.05		p <0.05		p > 0.05	
Roraima (RR)	38.24	23.77	55.88	54.77	52.94	9.95	5.88	6.76
	(13.64–62.83)	(2.23–45.31)	(30.75–81.01)	(29.58–79.96)	(27.68–78.20)	(0.00–25.10)	(0.00–17.79)	(0.00–19.47)
	p > 0.05		p > 0.05		p < 0.05		p > 0.05	
Pará (PA)	39.56	36.13	34.43	40.34	40.29	7.69	5.13	5.57
	(31.57–47.55)	(28.28–43.98)	(26.67–42.19)	(32.32–48.35)	(32.28–48.01)	(3.34–12.04)	(1.53–8.73)	(1.82–9.32)
	p > 0.05		p > 0.05		p < 0.05		p > 0.05	
Amapá (AP)	43.55	28.99	59.68	55.16	51.61	10.16	9.68	9.97
	(19.26–67.84)	(6.76–51.22)	(35.64–83.72)	(30.79–79.53)	(27.12–76.10)	(0.00–24.96)	(0.00–24.16)	(0.00–24.65)
	p > 0.05		p > 0.05		p < 0.05		p > 0.05	
Tocantins (TO)	8.89	21.57	51.11	43.03	33.33	9.08	15.56	11.29
	(4.16–13.62)	(14.73–28.41)	(42.80–59.42)	(34.80–51.26)	(25.49–41.17)	(4.30–13.86)	(9.53–21.59)	(6.03–16.55)
	p < 0.05		p > 0.05		p < 0.05		p > 0.05	
Northeast Region								
Maranhão (MA)	32.65	32.1	38.78	51.27	39.46	7.55	10.2	7.37
	(26.41–38.89)	(25.89–38.31)	(32.30–45.26)	(44.62–57.92)	(32.95–45.63)	(4.03–11.06)	(6.17–14.23)	(3.89–10.85)
	p > 0.05		p < 0.05		p < 0.05		p > 0.05	
Piauí (PI)	37.5	31.61	25	31.01	50	9.91	3.13	9.87
	(31.16–43.84)	(25.12–37.70)	(19.33–30.67)	(24.95–37.07)	(43.45–56.55)	(6.00–13.82)	(0.85–5.41)	(5.96–13.78)
	p > 0.05		p > 0.05		p < 0.05		p < 0.05	
Ceará (CE)	34.57	26.43	37.55	43.38	58.36	12.2	7.25	7.61
	(27.30–41.44)	(20.6–32.80)	(30.55–44.55)	(36.22–50.54)	(51.65–65.48)	(7.47–16.93)	(3.50–10.99)	(3.78–11.44)
	p > 0.05		p > 0.05		p < 0.05		p > 0.05	
Rio Grande do Norte (RN)	31.48	30.76	30.86	38.98	51.23	12.3	35.19	25.61
	(24.44–38.52)	(23.76–37.76)	(23.85–37.87)	(31.58–46.38)	(43.65–58.81)	(7.32–17.28)	(27.95–42.43)	(18.99–32.23)
	p > 0.05		p > 0.05		p < 0.05		p > 0.05	
Paraíba (PB)	35.79	32.3	32.11	40.97	47.89	11.89	11.05	11.36
	(29.50–42.08)	(26.16–38.44)	(25.98–38.24)	(34.52–47.42)	(41.33–54.45)	(7.64–16.14)	(6.94–15.16)	(7.20–15.52)
	p > 0.05		p > 0.05		p < 0.05		p > 0.05	
Pernambuco (PE)	32.76	30.82	41.81	48.97	49.39	11.16	23.96	18.57
	(25.98–39.54)	(24.15–37.49)	(34.68–48.94)	(41.75–56.19)	(42.17–56.61)	(6.61–15.71)	(17.79–30.13)	(12.95–24.19)
	p > 0.05		p > 0.05		p < 0.05		p > 0.05	
Alagoas (AL)	36.88	39.29	34.04	37.34	51.06	9.37	8.51	6.55
	(27.52–46.24)	(29.81–48.77)	(24.84–43.24)	(27.95–46.73)	(41.36–60.76)	(3.71–15.02)	(3.09–13.93)	(1.75–11.35)
	p > 0.05		p > 0.05		p < 0.05		p > 0.05	
Sergipe (SE)	48.05	36	40.26	45.3	56.49	12.75	15.58	9.74
	(36.74–59.35)	(25.14–46.86)	(29.16–51.36)	(34.03–56.57)	(45.27–67.10)	(5.20–20.30)	(7.37–23.79)	(3.03–16.45)
	p > 0.05		p > 0.05		p < 0.05		p > 0.05	
Bahia (BA)	33.75	29.66	38.04	49.4	56.68	12.56	8.31	7.2
	(29.21–38.29)	(25.28–34.04)	(33.80–42.70)	(44.60–54.20)	(51.92–61.44)	(9.38–15.74)	(5.66–10.96)	(4.72–9.68)
	p > 0.05		p < 0.05		p < 0.05		p > 0.05	
Southeast Region								
Minas Gerais (MG)	20.47	19.52	31.42	43.44	58.02	15	37.47	28.96
	(17.76–23.18)	(16.86–22.18)	(28.30–34.53)	(40.11–46.77)	(54.71–61.33)	(12.60–17.40)	(34.22–40.72)	(25.91–32.00)
	p > 0.05		p < 0.05		p < 0.05		p < 0.05	
Espírito Santo (ES)	15	21.89	34.55	41.76	53.64	13.81	35.45	23.65
	(11.51–29.42)	(10.72–28.32)	(23.99–45.10)	(30.82–52.70)	(42.57–64.71)	(6.15–21.47)	(24.83–46.07)	(14.22–33.08)
	p > 0.05		p > 0.05		p < 0.05		p > 0.05	
Rio de Janeiro (RJ)	22.52	21.95	53.39	64.63	55.91	14.59	37.28	30.77
	(13.98–31.06)	(13.49–30.41)	(43.20–63.58)	(54.86–74.40)	(45.76–66.06)	(7.38–21.80)	(27.40–47.16)	(21.34–40.20)
	p > 0.05		p > 0.05		p < 0.05		p > 0.05	
São Paulo (SP)	13.99	12.78	36.33	44.02	60.77	16.16	51.61	46.78
	(11.31–16.67)	(10.20–15.36)	(32.62–40.04)	(40.19–47.85)	(57.00–54.54)	(13.32–19.00)	(47.75–55.47)	(42.93–50.63)
	p > 0.05		p < 0.05		p < 0.05		p > 0.05	
South Region								
Paraná (PR)	23.27	19.46	40.45	42.62	56.18	13.66	60.51	61.77
	(19.12–27.42)	(15.58–23.34)	(35.63–45.27)	(37.77–47.47)	(51.31–61.05)	(10.29–17.03)	(55.71–65.31)	(57.00–66.54)
	p > 0.05		p > 0.05		p < 0.05		p > 0.05	
Santa Catarina (SC)	20.86	18	33.14	37.92	60.23	14.73	70.78	73.08
	(16.22–25.50)	(13.62–22.38)	(27.77–38.51)	(32.38–43.46)	(54.65–65.81)	(10.69–18.77)	(65.59–75.97)	(68.02–78.14)
	p > 0.05		p > 0.05		p < 0.05		p > 0.05	
Rio Grande do Sul (RS)	23.63	21.85	42.9	53.18	62.3	16.67	69.26	72.69
	(19.86–27.36)	(18.22–25.48)	(38.55–47.25)	(48.79–57.57)	(58.04–66.56)	(13.39–19.95)	(65.20–73.32)	(68.77–76.61)
	p > 0.05		p < 0.05		p < 0.05		p > 0.05	
Midwest Region								
Mato Grosso do Sul (MS)	21.43	23.25	54.76	54.05	46.43	11.07	30.95	30.69
	(12.38 – 30.4 )	(13.93 – 32.57)	(43.78 – 65.74)	(43.06 – 65.04)	(35.43 – 57.43)	(4.15 – 17.99)	(20.76 – 41.14)	(20.52 – 40.86)
	p > 0.05		p > 0.05		p < 0.05		p > 0.05	
Mato Grosso (MT)	17.16	17.27	33.58	36.87	57.46	9.95	23.13	22.92
	(10.94 – 23.38)	(11.03 – 23.51)	(25.78 – 41.38)	(28.91 – 44.83)	(49.3 – 65.62)	(5.01 – 14.89)	(16.17 – 30.09)	(15.98 – 29.86)
	p > 0.05		p > 0.05		p > 0.05		p > 0.05	
Goiás (GO)	20.23	18.6	39.31	42.82	43.35	11.18	23.84	22.74
	(15.29 – 25.35)	(13.74 – 23.46)	(33.21 – 45.41)	(36.64 – 49)	(37.16 – 49.54)	(7.24 – 15.12)	(18.52 – 29.16)	(17.5 – 27.98)
	p > 0.05		p > 0.05		p < 0.05		p > 0.05	
Federal District (DF)	15.24	16.1	35.24	44.18	59.05	18.32	27.62	21.2
	*	*	*	*	*	*	*	*
	*	*	*	*	*	*	*	*
Bazil	23.48	22.96	38.45	46.22	56.4	13.19	38.86	30.08
	(22.37 – 24.59)	(21.86 – 24.06)	(37.17 – 39.73)	(44.91 – 47.5 )	(55.1 – 57.7)	(12.3 – 14.08)	(37.58 – 40.14)	(28.88 – 31.28)
	p > 0.05		p < 0.05		p < 0.05		p < 0.05	

p < 0.05. Z-test was used for comparison between the Down and No Down groups.

Source: SINASC.

The prevalence of Down syndrome was significantly lower in Brazil among mothers with insufficient prenatal care, although there was no statistical significance between the states (Table 4). The difference between the Down syndrome and no Down syndrome groups was not affected by vaginal delivery, which was not significant in most of the states; however, the prevalence was high in Piauí in the Northeast region and low in Minas Gerais and São Paulo in Southeast region (Table 4). The prevalence of Down syndrome was significantly lower among mothers who delivered vaginally, although no significant differences were observed among the states (Table 4).

**Table 4:** Prevalence of prenatal care and vaginal delivery birth of mothers from 2010 to 2020 by states in Brazil.

Federative unit (Abbreviation)/Region	Mothers with insufficient prenatal care		Vaginal delivery birth	
	Down %	No Down %	Down %	No Down %
	(CI95%)	(CI95%)	(CI95%)	(CI95%)
	p-value		p-value	
North Region				
Rondônia (RO)	51.72	39.95	34.48	33.89
	(38.14–65.3)	(26.64–53.26)	(21.56–47.40)	(21.02–46.76)
	p > 0.05		p > 0.05	
Acre (AC)	56.67	58.16	58.33	61.14
	(35.96–77.37)	(37.54–78.77)	(37.73–78.93)	(40.77–81.51)
	p > 0.05		p > 0.05	
Amazonas (AM)	64.04	57.48	55.62	62.02
	(52.09–75.99)	(45.17–69.79)	(43.25–67.99)	(49.94–74.10)
	p > 0.05		p > 0.05	
Roraima (RR)	58.82	57.84	47.06	64.96
	(33.91–83.73)	(32.85–82.83)	(21.80–72.32)	(40.82–89.10)
	p > 0.05		p > 0.05	
Pará (PA)	55.68	55.39	48.35	51.5
	(47.57–63.79)	(47.27–63.51)	(40.19–56.51)	(43.34–59.66)
	p > 0.05		p > 0.05	
Amapá (AP)	64.52	63.8	66.13	65.73
	(41.08–87.96)	(40.25–87.35)	(42.94–89.32)	(42.44–88.96)
	p > 0.05		p > 0.05	
Tocantins (TO)	35.56	39.9	48.89	48.52
	(27.60–43.52)	(31.76–48.04)	(40.58–57.20)	(40.19–56.81)
	p > 0.05		p > 0.05	
Northeast Region				
Maranhão (MA)	55.1	60.52	48.98	56.56
	(48.48–61.72)	(54.02–64.02)	(42.33–55.63)	(49.60–63.10)
	p > 0.05		p > 0.05	
Piauí (PI)	46.88	44.2	62.5	46.89
	(40.34–53.42)	(37.70–50.70)	(56.16–68.84)	(40.35–53.43)
	p > 0.05		p < 0.05	
Ceará (CE)	39.78	33.86	40.52	43.54
	(32.71–46.85)	(27.02–40.70)	(33.43–47.61)	(36.38–50.70)
	p > 0.05		p > 0.05	
Rio Grande do Norte (RN)	33.33	38.88	31.48	41.08
	(26.18–40.48)	(31.49–46.27)	(24.44–38.52)	(33.55–48.47)
	p > 0.05		p > 0.05	
Paraíba (PB)	36.84	33.38	43.68	42.82
	(30.51–43.17)	(27.19–39.57)	(37.17–50.19)	(36.33–49.31)
	p > 0.05		p > 0.05	
Pernambuco (PE)	39.85	36.86	41.81	48.42
	(32.78–46.92)	(29.89–43.83)	(34.68–48.94)	(41.20–55.64)
	p > 0.05		p > 0.05	
Alagoas (AL)	48.94	45.9	46.1	45.71
	(39.24–58.64)	(36.23–55.57)	(36.43–55.77)	(36.04–55.38)
	p > 0.05		p > 0.05	
Sergipe (SE)	40.91	45.68	50	57.89
	(29.78–52.04)	(34.41–56.95)	(38.68–61.32)	(46.72–69.06)
	p > 0.05		p > 0.05	
Bahia (BA)	43.32	45.18	47.1	56.7
	(8.56–48.08)	(40.40–49.96)	(42.31–51.89)	(51.94–61.46)
	p > 0.05		p < 0.05	
Southeast Region				
Minas Gerais (MG)	22.03	25.18	34.03	42.64
	(19.25–24.81)	(22.27–28.10)	(30.85–37.21)	(39.32–45.96)
	p > 0.05		p < 0.05	
Espírito Santo (ES)	25.91	32.35	30.45	36.8
	(16.19–35.63)	(21.97–42.73)	(20.24–40.66)	(26.10–47.50)
	p > 0.05		p > 0.05	
Rio de Janeiro (RJ)	29.13	31.08	29.92	39.94
	(19.85–38.41)	(21.62–40.54)	(20.56–39.28)	(29.93–49.95)
	p > 0.05		p > 0.05	
São Paulo (SP)	22.66	21.54	30.74	40.17
	(19.43–25.89)	(18.37–24.71)	(27.18–34.30)	(36.39–43.95)
	p > 0.05		p < 0.05	
South Region				
Paraná (PR)	19.74	17.58	33.39	38
	(15.83–23.65)	(13.85–21.31)	(28.76–38.02)	(33.24–42.76)
	p > 0.05		p > 0.05	
Santa Catarina (SC)	30.02	26.14	38.21	41.22
	(24.79–35.25)	(21.13–31.15)	(32.67–43.75)	(35.60–46.84)
	p > 0.05		p > 0.05	
Rio Grande do Sul (RS)	25.82	25.02	32.92	38.04
	(21.97–29.67)	(21.21–28.83)	(28.79–37.05)	(33.77–42.31)
	p > 0.05		p > 0.05	
Midwest Region				
Mato Grosso do Sul (MS)	30.95	33.54	28.57	39.29
	( 20.76 – 41.14 )	( 23.13 – 43.95 )	( 18.61 – 38.53 )	( 28.52 – 50.06 )
	p > 0.05		p > 0.05	
Mato Grosso (MT)	27.61	31.16	35.82	39.39
	( 20.23 – 34.99 )	( 23.52 – 38.8 )	( 27.91 – 43.73 )	( 31.33 – 47.45 )
	p > 0.05		p > 0.05	
Goiás (GO)	28.32	33.23	27.17	33.71
	( 22.69 – 33.95 )	( 27.34 – 39.12 )	( 21.61 – 32.73 )	( 27.80 – 39.62 )
	p > 0.05		p > 0.05	
Federal District (DF)	24.76	28.66	29.52	45.55
	*	*	*	*
	*	*	*	*
Brazil	30.64	33.33	36.12	44.31
	( 29.43 – 31.85 )	( 32.09 – 34.57 )	( 34.86 – 37.38 )	( 43.01 – 45.61)
	p < 0.05		p < 0.05	

p < 0.05. Z-test was used for comparison between the Down and No Down groups.

Source: SINASC.

## Discussion

Brazil’s Sinasc allows for the collection of birth data across the country, providing information on all facets of public health.^13,16^ In the current study, data on Down syndrome births in Brazil were sampled, and the findings revealed that during the 10-year period between 2010 and 2020, there was an average of 30.4 newborns with Down syndrome born in Brazil for every 100,000 live births. Down syndrome was associated with low birth weights, ethnicity, and prematurity; however, there were fewer males with Down syndrome. Regarding the mother-associated factors, mothers aged 35 years and older and considered White had an increased prevalence of babies born with Down syndrome, while mothers without a spouse, insufficient prenatal care, and vaginal delivery had the lowest prevalence of newborns with Down syndrome. Although there was no difference among the regions in Brazil during the 10-year period analyzed, the South of Brazil had the highest prevalence of children with Down syndrome, while the Northeast, North, and Midwest regions had lower incidence. Santa Catarina and Maranhão had the highest and lowest prevalence of newborns with Down syndrome among states in Brazil, respectively.

To quantify the magnitude of Down syndrome prevalence precisely, it is imperative to conduct research focused on a specific country. Highly affluent nations are more able to collect precise data on patients with Down syndrome required for the national health surveillance.^3,12^ The Brazilian SINASC system used in this study provides the country’s statistics accessible to everyone and researchers, enabling comparisons between many Brazilian states.^14^ Brazilian authors have recently focused on the prevalence of Down syndrome.^11^ Contrarily, the research conducted by Laignier et al was retrospective and was carried out using secondary data included in the Certificate of Live Birth in a state located in the southeastern region of Brazil,^11^ rather than using Sinasc data system or covering the entire nation.

Down syndrome is the most frequent genetic cause of intellectual disability, and its incidence and occurrence differ according to the population.^10^ In the United States of America, the live birth prevalence of Down syndrome during the period 2006-2010 was estimated at 126 per 100,000 live births.^12^ In Southeast of Brazil, the prevalence of Down syndrome was estimated to be 40 per 100,000 live births during the period 2012–1018.^11^ In this study, the prevalence rate of Down syndrome during the 10-year period 2010–2020 in Brazil was 30.4 children per 100,000 live births.

Our findings revealed higher prevalence when compared to international research such as a Mexican study showing a prevalence of 3.73 per 100,000 live births^17^ and an Argentine study showing 17.26 per 100,000 live births.^18^ Our data were extracted from SINASC, which was implemented in the early 1990s. This was different from the study of Sierra Romero et al.^17^ where data were collected during the first four years after the Ministry of Health implemented an information system for live newborns, which serves as a national registry for those born with Down syndrome and other conditions. In this way, the Brazilian National Health Control could collect, with greater accuracy, the data of patients with Down syndrome compared to the Mexican system, with possibly underrated data. Among the Brazilian regions, the South region had the highest prevalence rate of Down syndrome, while the Northeast, North, and Midwest regions had the lowest rates. The average in the Southeast was 32.95 children with Down syndrome per 100,000 live births. This finding is different from what Laignier et al.^11^ obtained in the southeastern region, with an average of 4 per 100,000 live births from 2012 to 2018.

Down syndrome was evaluated in relation to the age of the mother and father, prematurity, degree of maternal education, low birth weight, and number of prenatal visits.^11,18^ Our findings demonstrated an association with maternal age, inadequate prenatal care, low birth weight, and preterm birth similar to other Brazilian and worldwide investigations.^11,18^

Conversely, our study only identified a relationship with maternal education in the state of Tocantins in the northern region, unlike the study carried out by Laigner et al.^11^ which established an association with maternal education in Brazil’s Southeast region. According to our study, parental education did not influence the incidence of Down syndrome nationally. However, education may play a significant role as a crucial preventative measure, particularly regarding the expected genetic effects of consanguinity.^11^

Additionally, our study found an association between the prevalence of Down syndrome and ethnicities at the national level. Regarding the states that showed statistical discrepancies among children with Down syndrome included Piauí in the Northeast region with a low prevalence, whereas Rio Grande do Norte (Northeast region), Minas Gerais (Southeast region), and São Paulo (Southeast region) had a higher proportion of White descent with Down syndrome. The differences in prevalence of children with Down syndrome were evaluated with mothers considered as White revealed that Minas Gerais (Southeast region) had a high prevalence rate, while it was low in Piauí (Northeast region). The highly varied genetic admixture of Brazilian people may affect the signs of Down syndrome and enhance their vulnerability.^19,20^ The huge ethnic heterogeneity of Brazil, which includes a wide variance in the proportions of ethnic groups within the population, may explain why our study found this association at the national level.^21^ Studies have shown that the prevalence of Down syndrome is two-fold higher among offspring of foreign-born mothers compared to those born to mothers native to the United States, particularly within non-Hispanic black and Hispanic populations.^19,20^ In accordance with the findings of this study, infants born to non-Hispanic black mothers showed a notably higher birth prevalence of Down syndrome compared to infants born to non-Hispanic white mothers.^20^

Males predominated the group of children born with Down syndrome. Our findings are similar to those of international investigation.^22^ This study revealed that there were disproportionately more boys than girls among children with a confirmed diagnosis of Down syndrome.^22^ The presence of more males among live births with non-mosaic trisomy 21 is consistent with data from other studies, suggesting that selection against female fetuses may be the cause.^22,23^ The prevalence of female mosaic trisomy carriers has been suggested to be due to sex-specific chromosomal losses during early embryogenesis.^22^

Regarding prenatal care, our study revealed a noteworthy nationwide statistical difference; however, this variation was not observed when examining individual states. Based on our findings, pregnant women expecting babies with Down syndrome were more likely to have six or more prenatal consultations compared to pregnant women expecting babies without Down syndrome.^11^ Technological breakthroughs in prenatal diagnosis, enabling the identification of Down syndrome within intrauterine life, coupled with the widespread legalization of abortion across many nations, have precipitated a growing trend wherein an increasing number of women opt to conclude their pregnancies following a diagnosis of trisomy 21.^12,24^ In Brazil, abortion is prohibited except in cases of anencephaly, sexual violence, or high risk to the pregnant woman’s life. Thus, in Brazil, the meticulous provision of prenatal care is fundamental for women carrying a fetus diagnosed with Down syndrome. This provide parents with essential information regarding their baby’s health in advance, allowing them to prepare emotionally for potential outcomes. A public reference hospital in Brazil showed that although 98% of mothers who participated in the study received prenatal care, only 4% obtained a prenatal diagnosis of Down syndrome.^8^

The main limitation of this study was its reliance on a secondary database. Despite being a national database in operation since 1990, it is subject to limitations related to form-completion errors and data-entry inconsistencies. Additionally, this study used information from SINASC data system, which depends on the declarations of live births. Errors or omissions in these declarations and subsequent data processing can lead to incomplete or inaccurate data. Further studies should delve deeper into the database and examine the relationship between data on individuals with Down syndrome and information on services, primary care coverage, and maternal/prenatal monitoring. By exploring all available variables thoroughly, these studies can enhance the quality of life of individuals with Down syndrome and their families.

Comprehensive care for people with Down syndrome is challenging, especially in vast countries such as Brazil. Understanding some of the epidemiological features of Down Syndrome could help with the implementation of health programs designed exclusively for these individuals.

## Conclusion

In Brazil, there were averagely 30.4 cases of Down syndrome for every 100,000 live births between 2010 and 2020, with no difference among the regions. Children with Down syndrome were more likely to be born to women aged > 35 years and older in Brazil. Additionally, Down syndrome was associated to ethnicity, insufficient prenatal care, and delivery methods. Individuals born with Down syndrome presented with low birth weight, preterm birth, and gender/ethnical differences. The findings of this study could assist the Brazilian healthcare system in strategic planning to ensure the enduring fiscal sustainability of individuals with Down syndrome and their mothers.

## Data Availability

The datasets generated during and/or analyzed during the current study are available from the corresponding author on reasonable request.
